# Polymorphisms in the *TOLLIP* Gene Influence Susceptibility to Cutaneous Leishmaniasis Caused by *Leishmania guyanensis* in the Amazonas State of Brazil

**DOI:** 10.1371/journal.pntd.0003875

**Published:** 2015-06-24

**Authors:** Felipe Jules de Araujo, Luan Diego Oliveira da Silva, Tirza Gabrielle Mesquita, Suzana Kanawati Pinheiro, Wonei de Seixas Vital, Anette Chrusciak-Talhari, Jorge Augusto de Oliveira Guerra, Sinésio Talhari, Rajendranath Ramasawmy

**Affiliations:** 1 Faculdade de Medicina, Universidade de Nilton Lins, Manaus, Amazonas, Brazil; 2 Fundação de Medicina Tropical Dr. Heitor Vieira Dourado, Manaus, Amazonas, Brazil; Institute of Tropical Medicine (NEKKEN), JAPAN

## Abstract

**Introduction:**

The clinical outcome to *Leishmania*-infection is determined by the individual adaptive immune T helper cell responses and their interactions with parasitized host cells. An early development of a proinflammatory immune response (Th1 response) is necessary for *Leishmania*-infection resolution. The Toll-interacting protein (TOLLIP) regulates human Toll-like receptors signaling pathways by down regulating the proinflammatory cytokines interleukin-6 (IL-6) and tumor necrosis factor alpha (TNF-α) and inducing the ant-inflammatory cytokine interleukin-10 (IL-10). Polymorphisms in the *TOLLIP* gene are associated with infectious diseases.

**Material and Methods:**

The polymorphisms rs5743899 and rs3750920 in the *TOLLIP* gene were genotyped by polymerase chain reaction and restriction fragment length polymorphism (RFLP) analysis in 631 patients with cutaneous leishmaniasis (CL) caused by *L*. *guyanensis* and 530 individuals with no history of leishmaniasis.

**Results:**

The G and T alleles of the rs5743899 and rs3750920 were more common in patients with CL than in healthy individuals (P = 2.6 x10^-8^ ; odds ratio [OR], 1.7 [ 95% confidence interval (CI) 1.4–2.0] and P = 1.9 x10^-8^ ; OR, 1.6 [95% CI 1.4–1.9] respectively). The r^2^ and D’ linkage disequilibrium between the two polymorphisms are 0.05 and 0.473 with a confidence bounds of 0.37 to 0.57 respectively.

**Conclusion:**

The two polymorphisms are independently associated with an increased risk of developing CL.

## Author Summary

Leishmaniasis is an infectious disease caused by the protozoan parasite of the *Leishmania* genus. It is transmitted by infected sandfly (the phlebotomines) and caused either visceral or tegumentary leishmaniasis depending on the species. In areas of endemicity for leishmaniasis, not all individuals exposed to the same environment develop the disease. It has been suggested that the genetic background of the individual may play a key role. The peri-rural regions of Manaus, the capital city of the Amazonas state of Brazil has become over the years endemic areas of mainly L. guyanensis American Tegumentary Leishmaniasis because of the constant deforestation and new settlements. Infected symptomatic patients most often develop localized cutaneous leishmaniasis (CL), characterized by a single or multiple well-demarcated cutaneous ulcer with raised borders and granulomatous center. In this study, we tried to understand why some individuals develop cutaneous leishmaniasis while other in the same area do not by genotyping two polymorphisms situated the TOLLIP gene and we saw that both polymorphisms are associated with cutaneous leishmaniasis.

## Introduction

Leishmaniasis, a vector-borne disease, is caused by the intracellular protozoan parasites of the *Leishmania* genus, which are transmitted by the phlebotomine sand fly. *Leishmania*-infection, depending on the *Leishmania* (*L*.) species and on the host specific immune response, presents a broad spectrum of clinical phenotypes ranging from asymptomatic, self-healing or non-healing cutaneous leishmaniasis (CL) to severe mucosal or life threatening visceral leishmaniasis (VL). Leishmaniasis affects nearly 12 million people, mostly in the tropical and subtropical countries, with a yearly incidence of more than 58,000 VL cases and 220,000 CL cases and is estimated to be the ninth largest infectious disease burden [1,2]. In Brazil, the estimated annual incidence of VL and CL cases is 4200 to 6300 and 72,000 to 119,000 respectively [2].

The major species that cause American tegumentary leishmaniasis (ATL) in Brazil are *L*. *braziliensis*, *L*. *guyanensis*, *L*. *lainsoni*, *L*. *amazonensis*, *L*. *shawi*, *L*. *naiffi* and *L*. *lindenbergi*. In the Amazonas, *L*. *guyanensis* is the main etiological agent of ATL [3]. Classical forms of ATL (cutaneous, disseminated, diffuse and mucosal leishmaniasis) are caused by *L*. *guyanensis*. The peri-rural regions of Manaus, the capital city of the Amazonas state, have become over the years areas of endemicities for ATL due to deforestation and new settlements.

The clinical outcome to *Leishmania*-infection is determined by the individual adaptive immune T helper cell responses and their interactions with parasitized host cells. An early development of a proinflammatory immune response (Th1 response) and nitric oxide (NO) generation is necessary for *Leishmania*-infection resolution [4]. Th1 responses are associated with healing and parasite killing, whereas Th2 responses are associated with non-healing diseases and uncontrolled parasite growth [5]. However, the host-pathogen interaction has several layers of complexity, including the genetic background of the host, the virulence and genotype of the parasite, the *Leishmania*-vector phlebotomine and an environment favorable for the development of lesions. In *L*. *braziliensis* endemic regions, subjects with a positive delayed type hypersensitivity test (DTH+) response indicating exposure to *Leishmania* antigen but lack a history or exam suggesting they had the disease *per se* are also common besides patients with CL or ML, raising the question of whether asymptomatic infection had occurred [6]. The clinical spectrum of the disease suggests that the host genetics may play an important role in the outcome. Familial aggregation of CL and ML in endemic regions argues for genetic susceptibility [7].

Several studies have shown the involvement of Toll-like receptors (TLR) in immunity to protozoan parasites [8,9]. TLRs are cited to play an important role in the immune response to *Leishmania*-infection [10,11]. TLR4-mediated induced nitric oxide synthase (iNOS) leads to increased intracellular *Leishmania*-killing [12]. Knockdown of TLR2, TLR3, IRAK1 and MyD88 expression by RNA interference lead to decrease secretion of NO and TNFα induced by *L*. *donovani* promastigotes [13]. Human NK cells are activated upon recognizing purified lipophosphoglucan from *Leishmania* through TLR2 [14]. TLR4 restrained parasite growth in both innate and adaptive immunity to *L*. *major* infection by the induction of iNOS [12]. Comparison of induced infections in C57BL10/ScN mice carrying a null mutation in TLR4 to TLR4-competent C57BL10/ScSn mice showed high parasite loads in the C57BL10/ScN mice [15]. MyD88, an adaptor protein in the signaling pathway of TLR, seems to be very important for the secretion of IL-1α by mouse peritoneal macrophages following infection by *L*. *major* [16]. MyD88-deficient mice are very susceptible to infection with *L*. *major* [17]. LPG from *L*. *major* stimulates macrophages to secrete IL-12 and TNFα via MyD88 and requires TLR2 to activate NfKB [17].

Increasing emerging evidence indicates a significant contribution of TLR4 in restraining the *Leishmania* multiplication in both innate and adaptive immune responses [18]. TLR4-mediated IL12 signaling is important for resistance against *Leishmania*. TLR2-deficient mice showed enhance resistance to *L*. *braziliensis* infection while MyD88 deficient mice are susceptible to the infection [19].

These studies suggest that the genes involved in the TLR pathway may play an important role in the susceptibility to leishmaniasis. Toll-interacting protein (TOLLIP) is a negative regulator in the TLR signaling cascade [20], particularly in the suppression of TLR2 and TLR4 pathway [21]. TOLLIP-deficient mice induced proinflammatory pathways [22].

IL-6 and tumor necrosis factor alpha was shown to be significantly reduced in TOLLIP-deficient mice after treatment at low doses with IL-1beta and LPS leading the authors to suggest that TOLLIP may control the magnitude of inflammatory cytokine production [22]. Recently, TOLLIP was suggested to regulate human TLR signaling pathways by inhibiting proinflammatory cytokines, particularly IL6 and TNFα, and inducing the anti-inflammatory cytokine IL-10 [23]. Interestingly, two variants (rs5743899 and rs3750920) of the *TOLLIP* gene associated with tuberculosis were shown to be associated with mRNA expression and TLR mediated cytokine release [23].

Taking into account that a balance Th1 response mediates parasite killing and the importance of the TLR pathway in restraining parasite multiplication in animal models, we investigated whether the polymorphisms rs5743899 associated with increased levels of IL-6 and rs3750920 with decreased levels of mRNA expression of the *TOLLIP* gene, a negative regulator of TLR4 and TLR2, may be associated with CL caused by *L*. *guyanensis* in the state of Amazonas, Brazil.

## Materials and Methods

### Study population

The present study was conducted in the peri-rural region of Manaus, the capital city of the Amazonas state, Brazil. Over the years there has been increasing deforestation for settlements, agriculture and farming. These areas have become endemic particularly for *L*. *guyanensis*. All of the case patients included in the study is patients with active CL (fewer than six lesions) from whom the parasites were isolated at the FMT-HVD, a referral hospital for treating leishmaniasis patients. Only patients infected with *L*. *guyanensis* were included in the study. All of the controls participating in the study are from the same endemic area sharing similar environments. Most of the participants are agricultural workers and has been living in the same area for more than ten years.

A total of 631 patients with CL confirmed by direct microscope examination of Giemsa-stained specimens for the presence of *Leishmania* parasites from lesion scarifications and 530 healthy controls with no history of leishmaniasis participated in the study. Basic characteristics of the study population are shown in Table 1. Of the 631 patients with CL, 492 (78%) were male with a mean age of 33±SD13.2 years and 139 (22%) were female (mean age 33±SD 15.4 years). Among the healthy controls, 326 (62%) were male with a mean age of 39±SD17.7 years and 204 (38%) were female (mean age 36±SD17.0 years). The difference between sex distributions between the patients with CL and healthy controls was statistically significant (p <0.05). Healthy controls were slightly older than patients with CL.

**Table 1:** Basic characteristics of the study population.

	Patients with CL		Controls	
	n:631		n:530	
	Males	Females	Males	Females
**SEX**	492 (78%)	139 (22%)	326 (62%)	204 (38%) *
**AGE**	33.34 ± 13.17	32.88 ± 15.38	39.15 ± 17.68	16.98 **

*Differences between genders are statistically significant p<0.05

** Healthy controls are slightly older than patients with CL (Cutaneous leishmania) p<0.05

### Ethics statement

This study was conducted according to the principles expressed in the declaration of Helsinki and was approved by the Research Ethics Committee of the Fundação de Medicina Tropical Dr. Heitor Vieira Dourado (FMT-HVD) granted under the file number CAAE:09995212.0.0000.0005. All of the participants or their responsible party for individuals less than 18 years old provided written informed consent for the collection of samples and subsequent analysis. The responsible party for individuals under 18 were either parents or guardians of the minor participants. This is a case-control study comparing unrelated patients with CL to healthy unrelated individuals as controls with no history of leishmaniasis coming from the same area of endemicity.

### *Leishmania* genotyping

DNA was prepared from lesion biopsy specimens of all the patients with CL. *Leishmania vianna* specific PCR with discrimination between *L*. *braziliensis* and *L*. *guyanensis* were performed according to protocols previously described elsewhere [24,25].

### DNA preparation from patients with CL and healthy controls

Blood (5 mL) was collected by venipuncture into EDTA- containing Vacutainers (Becton Dickinson). Genomic DNA was prepared according to the proteinase K and salting-out method [26].

### *TOLLIP* polymorphisms rs5743899 and rs3750920 genotyping by Polymerase Chain Reaction-Restriction Fragment Length Polymorphism (PCR-RFLP)

The polymorphisms rs5743899 and rs3750920 of the *TOLLIP* gene present in the intron and exon 4 respectively were typed by PCR-RFLP using the restriction enzymes *Hha I* for rs5743899 and *Msp I* for rs3750920 (New England Biolabs). The following pairs of primers: rs5743899F: 5’-GGC AAT GGC AGT GGC CAC CAG TGA-3’ and rs5743899R: 5’-CCG ATG CCC GCA CAC CTG TGT GAT-3’ for (rs5743899) and rs3750920F: 5’-AGG CGT GCA GCT CAC CGC GTA GGA-3’ and rs3750920R: 5’-GAG AGC CTT CTC CAT GGA CGA CCG C-3’ for (rs3750920) were used to amplify the regions encompassing the polymorphisms separately. PCR was performed in a final volume of 25μL containing 2.0 mmol/L MgCl_2,_ 0.2 pmol/L each of forward and reverse primer, 40 μmol/L each dNTP, 50 ng of DNA and 2 U of *Taq polymerase* in buffer containing 500mmol/L KCl and 100mol/L Tris-HCL (pH 8.3). For both polymorphisms, the PCR cycling conditions included an initial denaturation step of 5 min at 95ºC, followed by 40 cycles of 15 s at 95ºC, 15 s at 62ºC, 30 s at 72ºC and a final extension step for 7 min at 72ºC. The generated PCR fragments were 279bp for rs5743899 and 169bp for rs3750920. A volume of 10uL of the PCR products was digested with the corresponding restriction enzymes *Hha I* and *Msp I*. For rs5743899, when the allele G is present, the 279bp fragment is cleaved into 125, 93 and 61bp whereas for the allele A, the 279bp fragment generates 218 and 61bp. Similarly for rs3750920, when the allele T is present the 169bp fragment is cleaved into 117 and 52bp and remains uncleave for the allele C. PCR restriction fragments were size separated by electrophoresis in 3% agarose gel.

### Statistical analysis

Statistical analysis was performed using logistic regression analysis using the website http://ihg.gsf.de/cgi-bin/hw/hwa1.pl. Comparison between groups used a two-tailed χ2-test. The odds ratio (OR) with 95% confidence interval [CI] were estimated. Hardy–Weinberg expectation (HWE) was determined by comparing the observed number of different genotypes frequency with those expected under HWE. Linkage disequilibrium was performed with the software Haploview 4.2.

## Results

The genotypes and allele frequencies of rs5743899 and rs3750920 are shown in Table 2. There was no evidence of deviation from Hardy-Weinberg equilibrium for both polymorphisms in either groups of patients with CL or controls. Comparison of patients with CL to controls showed significant allele-wise and genotype-wise associations. Genotype distributions were different between patients with CL and controls (rs5743899, p = 5.2x10^-8;^ rs3750920, p = 2.9x10^-8^). There were an excess of genotypes of the rs5743899 G allele (AG + GG; 54%) and of the rs3750920 T allele (TT + CT; 64%) in the patients with CL group compared to 40% and 51% respectively in the control group. Individuals homozygous for rs5743899 G allele or rs3750920 T allele had three times risks of developing CL compared to individuals homozygous for the rs5743899 A allele (P = 1.2x10^-6^; OR, 3.0 [95% CI 1.9–4.8]) or rs3750920 C allele (P = 6.0x10^-8^; OR, 2.9 [95% CI 1.9–4.2]). In the dominant model, carriers of the rs5743899 G allele or rs3750920 T allele were compared with homozygous individuals for the A or C allele respectively and the difference were highly significant (P = 1.9x10^-6^ OR, 1.8 [95% CI 1.4–2.2] and (p = 5.4x10^-6^; OR, 1.7 [95% CI 1.4–2.2]). A similar trend was observed when heterozygous individuals for both polymorphisms (rs5743899 AG or rs3750920 CT) were compared to rs5743899 AA or rs3750920 CC homozygous individuals.

**Table 2:** Genotype and allele frequencies for the single nucleotide polymorphisms rs5743899 and rs3750920 in patients with cutaneous leishmania (CL) and healthy controls.

	Patients with CL	Controls
**rs5743899**	Total: 631	Total: 530
**Genotypes**		
**AA**	289 (46%)	317 (60%)
**AG**	265 (42%)	185 (35%)
**GG**	77 (12%)	28 (5%)
**Alelle**		
**A**	843 (67%)	819 (77%)
**G**	419 (33%)	241 (23%)
Genotypes and alleles comparisons		
	P value	OR [95% CI]
**GG vs. AA**	1.2 x10^-6^	3.0 [2.0–4.8]
**GG + AG vs. AA**	1.9 x10^-6^	1.8 [1.4–2.2]
**AG vs. AA**	0.0003	1.6[1.2–2.0]
**G vs. A**	2.5x10^-8^	1.7 [1.4–2.0]
**rs3750920**		
**Genotypes**		
**CC**	225 (36%)	259 (49%)
**CT**	294 (46%)	226 (43%)
**TT**	112 (18%)	45 (8%)
**Alelle**		
**C**	744 (59%)	744 (70%)
**T**	518 (41%)	316 (30%)
	P Value	OR [95% CI]
**TT vs. CC**	6.0 x10^-8^	2.9 [1.9–4.2]
**TT + CT vs. CC**	5.4x10^-5^	1.7 [1.4–2.2]
**CT vs. CC**	0.001	1.5 [1.2–1.9]
**T vs. C**	1.9 x10^-8^	1.6 [1.4–1.9]

As there was an excess of females in the controls group, we stratified the groups by sex and repeated the comparisons. As shown in the S1 Table the associations for both polymorphisms were independent of sex. For the rs5743899, distribution of genotypes in males and females with CL were statistically different compared to controls (p = 6.1x 10^−6^ and 3.0 x 10^−4^ respectively). When the patients were categorized as those who had the G allele (AG and GG) and those who did not (genotype AA), a statistically significant difference between the patients with CL and the controls was also found for both males and females (males; P = 4 x 10^−5^ and females; P = 1.7 x 10^−3^). Thus, the rs5743899 allele seems to confer susceptibility to CL. Differences in allele frequencies between the patients with CL and the controls were also statistically significant (males; P = 2.8x 10^−6^; OR, 1.7 [95% CI, 1.4–2.2] and females; P = 3.5 x 10^−4^ OR, 1.7 [95% CI, 1.4–2.2]). Similar trend was observed for the rs3750920.

The linkage disequilibrium (LD) between the two polymorphisms is very low as calculated by the Haploview 4.2 program. The r^2^ and D’ values of the LD are 0.05 and 0.0.473 with a confidence bounds of 0.37 to 0.57 respectively suggesting that the two SNPs are independently associated with CL.

## Discussion

Enormous headway has been achieved in the understanding of Leishmaniasis but the molecular mechanism that underlies the development of the clinical outcome still remains obscure. In endemic areas of tegumentary leishmaniasis, only a fraction of individuals infected by the *Leishmania* parasite develop CL and a very small proportion evolves to mucosal leishmaniasis. Increasing interest in the relationship between host genetics and the outcome of *Leishmania*-infection has emerged. The identification of genes involves in the susceptibility to *Leishmania*-infection may pave the way for a better understanding of the disease and for designing vaccine. Dysregulation and genetic polymorphisms in the immune system are associated to many infectious diseases.

The TLR family plays a key role in innate immunity and in bridging innate and adaptive immunity [27] Several genetic variations in the TLR-signaling pathway are associated with either susceptibility or resistance to several infectious diseases [28,29]. TLRs orchestrate innate immune response on recognition of invading pathogens, through the induction of chemokines and inflammatory cytokines. CL is linked to a predominantly type 1 immune response where the proinflammatory cytokines TNF-α and IFN-γ mediate macrophage activation to control the parasite [30,31]. In leishmaniasis, the regulation of the cellular immune response together with an appropriate balance between proinflammatory and anti-inflammatory cytokines is important to achieve cure and prevent immune-mediated pathological damage or reactivation of disease [32,33]. Inadequate counterregulation of proinflammatory cytokines may be responsible for the development of cutaneous lesions in *Leishmania*-infection.

The proinflammatory reaction elicited by TLRs needs to strike an appropriate balance, resulting in an inflammatory response suited to keeping the invading pathogens in checks; otherwise excess activation of the intracellular signaling pathways may lead to an exacerbation of proinflammatory cytokines such as TNF-α and IFN-γ and reactive nitrogen intermediates, which can result in host tissue damage. TOLLIP is a negative regulator of TLRs-signaling and two polymorphisms, rs5743899 and rs3750920, in the *TOLLIP* gene have been associated with tuberculosis in the Vietnamese population [23]. The current study showed that both polymorphisms are associated with CL.

In tuberculosis, the rs5743899 G allele was shown to confer susceptibility in a recessive model while the rs3750920 T allele is protective [23] in contrast to a study in leprosy where the T allele was predominant [34]. The observed association of the rs3750920 T allele to CL in this study is in line with the association with leprosy [34] Shah et al. showed that the rs5743899 GG genotype is associated with increased levels of the proinflammatory cytokine IL-6, low levels of IL-10 and decreased TOLLIP mRNA expression while the rs3750920 TT genotype was associated with high TOLLIP mRNA expression but with no correlation with the levels of either IL-6 or IL-10 [23]. Intralesional cytokines study from biopsy specimens of patients with active CL due to *L*. *guyanensis* differentiated patients into three groups [35]. Patients with a predominance of Th2 cytokines developed lesions earlier compared to patients with a predominance of Th1 cytokines while in patients with similar Th1 and Th2 cytokines, lesion development was intermediate between patients with Th1 and Th2 [35]. Pretreatment of macrophages with human recombinant IL-6 inhibits IFN-γ and TNF-α-mediated activation of macrophages for killing of *L*. *amazonensis* [36]. IL-6 has been shown to downregulate the expression of TNF-α membrane receptors [37]. It is plausible that individuals with high levels of IL-6 cannot control the parasites. The persistence of the parasite triggers ongoing inflammation leading to tissue damage. Furthermore, it has been shown that IL-6 produced by professional antigen presenting cells induced the expression of the nuclear factor of activated T cells promoting Th2 cells differentiations [38]. High expression of Th2 cytokines, IL4 and IL13, has been shown in biopsy specimens from patients with CL caused by *L*. *guyanensis* [35]. In *L*. *major-*infected mice, the development of CD^+^Th1 cells or Th2 cells leads to resistance or susceptibility to infection respectively [39].

Functional studies are needed to understand how these polymorphisms are correlated with high levels of TOLLIP mRNA expression or levels of cytokines and also to understand its association with susceptibility to CL caused by *L*. *guyanensis*. The rs5743899 is located in the second intron of the gene while the rs3750920 in the fifth exon with a synonymous change proline to proline. Neither of the polymorphisms would seem to have an impact on the gene function. It is highly possible that these polymorphisms may be in linkage disequilibrium with an as yet unidentified variant which might influence the level of transcription. Furthermore, the functional significance of these polymorphisms was done in a predominantly European-American population. In the present study, the population is mainly an admixture of Native American Ancestry with European ancestry and is called “Caboclo”. It has been shown that this population is of origin of nearly 50 to 60% of Native American, 40 to 50% European and around 10% African ancestry [40]. The minor allele frequencies (allele T) for the rs3750920 observed in this population 0.30 is nearly similar to the Vietnamese (0.35) [23], Asian (0.36) and African (0.33) but lower in comparison to Caucasia population (0.47) [HapMap CEU, Asian and YRI]. The minor allele frequencies of the rs5743899 (allele G) is 0.23 compared to 0.41 in the Vietnamese population. Further study would be needed to confirm the association with another larger subset of patients with CL. Moreover, a panel of single nucleotide polymorphisms covering the whole gene besides the two SNPs would be interesting to construct LD plot and compare to other populations. The association of the rs3750920 T and rs5743899 G allele to CL is not absolute to explain cutaneous leishmaniasis in patients as these alleles are also present in the healthy controls from the same endemic areas. However, these two alleles can be assumed to be one of the several other predisposing risk factors to C as this a complex diseases where several factors, both genetics and environmental, may act together to develop the disease.

Altogether, our present findings suggest that the polymorphisms in the *TOLLIP* gene are associated with CL caused by *L*. *guyanensis* and TOLLIP may play an important role in the immunopathogenesis of the disease. This data may shed new light on the pathogenic mechanism of the disease and raise a potentially interesting issue that is worthy of further study of these polymorphisms in patients with CL caused by other species of *Leishmania*.

## Supporting Information

S1 Table Genotype and allele frequencies of the rs5743899 and rs3750920 in the study population stratified by sex. (DOCX)
