# Supplementary material for: Polymorphisms in the TOLLIP Gene Influence Susceptibility to Cutaneous Leishmaniasis Caused by Leishmania guyanensis in the Amazonas State of Brazil
Source: PLoS Negl Trop Dis. 2015 Jun 24;9(6):e0003875. doi: 10.1371/journal.pntd.0003875 (PMC4479602; doi:10.1371/journal.pntd.0003875)
Supplement: S1 Table — (DOCX) [file pntd.0003875.s001.docx]

**S1 Table.** Genotype and Allele frequencies of the rs5743899 and rs3750920 in the study population stratified by sex.

CL: Cutaneous leishmania

|  | | Patients with CL, no. (%) | | | Controls, no. (%) | | | |
| --- | --- | --- | --- | --- | --- | --- | --- | --- |
|  | | **Males** | **Females** | | **Males** | **Females** | |  |
| rs5743899 | | **n= 492** | **n=139** | | **n=326** | **n=204** | |  |
| Genotypes | |  |  | |  |  | |  |
| AA | | 236 (48%) | 53(38%) | | 204 (63%) | 113(56%) | |  |
| AG | | 198 (40%) | 67(48%) | | 105 (32%) | 80(39%) | |  |
| GG | | 58 (12%) | 19(14%) | | 17 (5%) | 11(5%) | |  |
| Alelle | |  |  | |  |  | |  |
| A | | 670 (68%) | 173(62%) | | 513 (79%) | 306(75%) | |  |
| G | | 314 (32%) | 105(38%) | | 139(21%) | 102(25%) | |  |
| Genotypes and alleles comparisons | | | | | | | | |
|  | | **Males**  **P Value OR [95% CI]** | | | **Females**  **P Value OR [95% CI]** | | | |
| GG vs. AA | | 0.0001 3.0 [1.7 – 5.2] | | | 0.001 3.7 [1.6 – 8.3] | | | |
| GG + AG vs. AA | | 4.0 x10^-5^ 1.8 [1.4 - 2.4] | | | 0.001 2.0 [1.3 – 3.1] | | | |
| AG vs. AA | | 0.001 1.6 [1.2 - 2.2] | | | 0.01 1.8 [1.1– 2.8] | | | |
| G vs. A | | 2.8 x10^-6^  1.7 [1.4 - 2.1] | | | 3.5 x10^-4^  1.8 [1.3 – 2.5] | | | |
|  |  | | |  |  | |  | |
| rs3750920 |  | | |  |  | |  | |
| Genotypes |  | | |  |  | |  | |
| CC | 178 (36 %) | | | 47 (34%) | 144 (44%) | | 115 (56%) | |
| CT | 229 (47%) | | | 65 (47%) | 149 (46%) | | 77 (38%) | |
| TT | 85 (17%) | | | 27 (19%) | 33 (10%) | | 12 (6%) | |
| Alelle |  | | |  |  | |  | |
| C | 585 (59%) | | | 159 (57%) | 437 (67%) | | 307 (75%) | |
| T | 399 (41%) | | | 119 (43%) | 215 (33%) | | 101 (25%) | |
|  | | | | | | | | |
|  | | **Males** | | | **Females** | | | |
| TT vs. CC | | 0.001 2.1 [1.3 – 3.3] | | | 2.9 x10^-6^  5.5 [2.6 – 11.8] | | | |
| TT + CT vs. CC | | 0.02 1.4 [1.0 – 1.9] | | | 4 x10^-5^ 2.5 [1.6 – 3.9] | | | |
| CT vs. CC | | 0.1 1.2 [1.0 – 1.7] | | | 0.002 2.1 [1.3 – 3.0] | | | |
| T vs. C | | 0.001 1.4 [1.1 – 1.7] | | | 6.5 x10^-7^  2.0 [1.6 – 3.1] | | | |
